# Saudi Medical Appointments and Referrals Center (SMARC) Performance Dynamic: A Comparative National Analysis of 2023–2024 Against Baseline Metrics

**DOI:** 10.3390/healthcare13161945

**Published:** 2025-08-08

**Authors:** Abdullah A. Alharbi, Ahmad Y. Alqassim, Meshary S. Binhotan, Mohammed A. Muaddi, Ali K. Alsultan, Mohammed S. Arafat, Abdulrahman Aldhabib, Yasser A. Alaska, Eid B. Alwahbi, Aidrous M. Ali, Mohammed K. Alabdulaali, Nawfal A. Aljerian

**Affiliations:** 1 Family and Community Medicine Department, Faculty of Medicine, Jazan University, Jazan 45142, Saudi Arabia; aaalharbi@jazanu.edu.sa (A.A.A.); aalqassim@jazanu.edu.sa (A.Y.A.); 2 Emergency Medical Services Department, College of Applied Medical Sciences, King Saud bin Abdulaziz University for Health Science, Riyadh 11481, Saudi Arabia; hotanm@ksau-hs.edu.sa (M.S.B.); njerian@moh.gov.sa (N.A.A.); 3 King Abdullah International Medical Research Centre, Riyadh 11481, Saudi Arabia; 4 Medical Referrals Centre, Ministry of Health, Riyadh 12382, Saudi Arabia; alkalsultan@moh.gov.sa (A.K.A.); arafatms@moh.gov.sa (M.S.A.); aaldhabib@moh.gov.sa (A.A.); ealwahbi@moh.gov.sa (E.B.A.); amagzoub@moh.gov.sa (A.M.A.); 5 Department of Emergency Medicine, King Saud University, Riyadh 11461, Saudi Arabia; yalaska@ksu.edu.sa; 6 Ministry of Health, Riyadh 12382, Saudi Arabia; mal-abdul-aali@moh.gov.sa

**Keywords:** e-referral systems, healthcare coordination, access to care, Saudi Arabia’s Vision 2030, performance metrics, healthcare access

## Abstract

**Background/Objectives:** Saudi Arabia implemented the Saudi Medical Appointments and Referrals Centre (SMARC) e-referral system to coordinate patient transfers and enhance healthcare access across the country. This nationwide system was established to improve coordination between healthcare facilities and provide timely access to specialized services. SMARC operates as a centralized coordination hub connecting secondary and tertiary care facilities across all specialties nationwide. This study evaluates SMARC’s evolution since 2020–2021 and efficiency improvements through 2023–2024 after major expansion efforts. **Methods:** This retrospective analysis examined 755,145 e-referrals across all 13 administrative regions of Saudi Arabia during 2023–2024. The study analyzed data extracted from the SMARC database covering two consecutive years. Outcomes assessed included acceptance rates, referral destinations (internal within the same region vs. external to other regions), and factors associated with system performance. **Results:** The total volume of e-referrals through SMARC increased substantially by 19.34% to 755,145 in 2023–2024. Acceptance rates for referrals improved markedly from 74.13% to 90.19% over this period. The proportion of internal referrals increased from 80.13% of total referrals to 87.52%. In contrast, external referrals to other regions declined from 19.87% to 12.48% of the total. Critical care referrals (ICU, CCU, NICU, PICU) decreased from 12.39% to 9.91%. Referrals for life-saving emergency conditions showed a noticeable decrease from 6.65% to 2.18%. Referrals to hospital outpatient departments (OPD) also showed an increase from 48.07% to 66.66% of total referrals. **Conclusions:** SMARC demonstrated considerable improvements in key metrics including referral acceptance rates and growth in regional self-sufficiency. This progress is associated with the Kingdom’s goals for advancing its healthcare system under Vision 2030 initiatives. The system has enabled more effective care coordination and access to specialized services across regions. These achievements were observed during a period of significant healthcare infrastructure expansion documented during this period, including growth in specialized centers, increased ICU bed capacity following governmental regulation after the COVID-19 pandemic, and expansion of trained medical subspecialists.

## 1. Introduction

Electronic referral (e-referral) systems have emerged globally as critical infrastructure for coordinating patient transfers between healthcare facilities, demonstrating benefits in quality of care, specialized service access, and resource optimization [1]. E-referrals reduce waiting times, decrease administrative burden, improve data completeness, and enhance provider communication across different care levels [1,2]. Despite these advantages, implementation challenges persist, including technological integration barriers, inconsistent provider adoption, clinical information standardization problems, and governance framework sustainability issues [2,3]. High-performing systems provide valuable implementation benchmarks, such as Denmark’s system with nearly universal adoption among general practitioners and documented cost savings [4], and Catalonia’s electronic health information exchange enabling critical data sharing [5]. Emerging evidence suggests standardized communication pathways significantly benefit referral management [3], while artificial intelligence (AI) tools show promise for improving triage decision-making accuracy. Digital health innovations, including telemedicine and electronic health records, represent essential components in healthcare transformation strategies, particularly for connecting geographically diverse provider networks [6].

The Saudi healthcare system is ranked 26th among 191 countries globally, with the Ministry of Health (MoH) currently controlling over 60% of healthcare facilities, while private and other public sectors represent the remaining 40% [7]. This government-led model with significant public sector involvement is complemented by a substantial private healthcare presence [8]. As a country with a rapidly developing healthcare system, Saudi Arabia faces mounting pressure on its healthcare infrastructure, driving a shift toward privatization [9]. In alignment with Vision 2030, the country has initiated a transition toward increased privatization of healthcare services [10]. The Saudi Medical Appointments and Referrals Centre (SMARC) represents a cornerstone of Saudi Arabia’s healthcare transformation strategies under Vision 2030, functioning as a centralized coordination hub for patient transfers between healthcare facilities nationwide [6,11]. Initially launched as “Ehalati” in 2012, SMARC underwent significant expansion to address fragmented hospital systems and limited cross-sector integration [12]. Unlike other e-referral models where referrals originate from primary healthcare to specialized care or between two specialized care facilities that are either hospital-based or regional based systems [1,13,14,15], the SMARC e-referral system organizes referrals between secondary healthcare levels and beyond, encompassing all specialties nationwide. It employs a digital platform integrating all MoH hospitals, government hospitals, and the majority of private hospitals to streamline the referral process. This centralized system offers a network pool of hospitals to efficiently provide access to the necessary healthcare resources across the regions of Saudi Arabia. Operating within the Kingdom’s healthcare structure serving nearly 32 million people [16], SMARC supports the strategic reorganization of Saudi Arabia’s 13 administrative regions into five business units (BUs): Central, Eastern, Western, Northern, and Southern [11,17]. This reorganization aligns with Vision 2030’s healthcare objectives of boosting quality, efficiency, and value through integrating public and private sectors under MoH oversight [17,18,19]. Building upon baseline performance assessments from 2020 to 2021, SMARC implemented substantial enhancements during 2020–2024, including the introduction of offsite services modality as an alternative to full patient transfers. The system’s effectiveness lies in its ability to connect secondary and tertiary care facilities through a unified digital infrastructure that manages the entire referral workflow, from request submission to transfer coordination, ultimately improving healthcare access and resource utilization across geographical boundaries [11].

While previous studies examined SMARC’s baseline e-referral performance in 2020–2021, documenting acceptance rates of 74.13% [12] and external referral rates of 19.87% [3], this research evaluates its evolution and efficiency improvements from baseline to 2023–2024. This addresses a significant knowledge gap by providing insights into healthcare system transformation trajectories. This study aims to characterize referral patterns and performance metrics over time using data from SMARC. We alternatively hypothesized that there is significant improvement in SMARC performance metrics, including referral volumes, acceptance rates, and others, between 2020–2021 and 2023–2024. The analysis examined regional variations, key performance indicators, and progress toward healthcare self-sufficiency across different parts of the country. Regional self-sufficiency is defined as the percentage of referrals managed internally within each region, with higher internal referral rates indicating greater local healthcare capacity to meet patient needs. The overall goal was to identify factors associated with referral practices and outcomes in order to inform efforts to optimize the e-referral system and advance healthcare transformation initiatives aligned with national objectives [6,11,17]. This study provides evidence for resource allocation policies by quantifying SMARC’s performance improvements across diverse regions. Measurable transformation progress is demonstrated through increased acceptance rates and expanded internal referral capacity [6,17]. These quantifiable indicators establish benchmarks for evaluating initiatives and guiding optimization strategies. Additionally, SMARC’s enhanced workflows offer transferable knowledge for developing e-referral systems [20,21].

## 2. Materials and Methods

### 2.1. Study Design, Setting, and Data Collection

**Figure 1:**
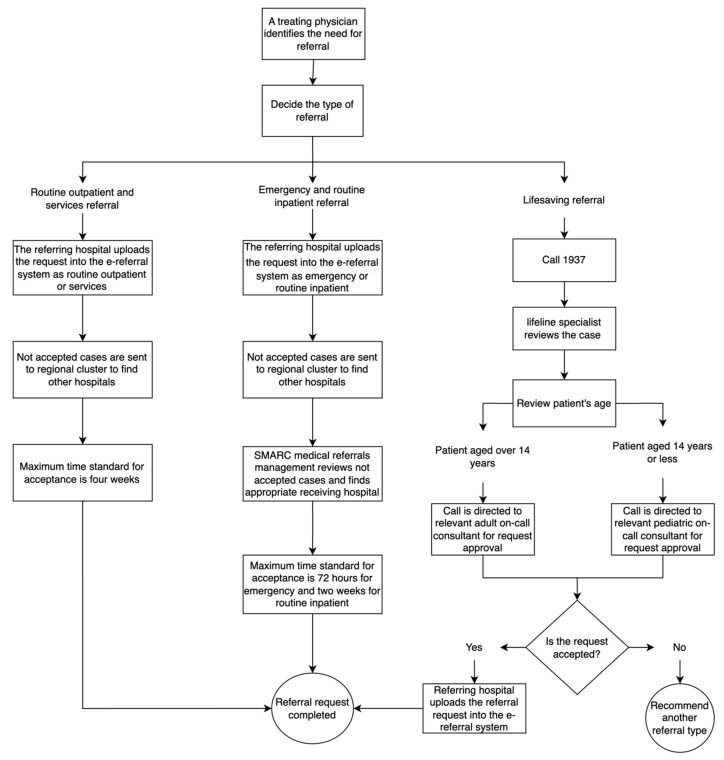
The Process of Requesting a Referral Through the Saudi Medical Appointments and Referrals Center (SMARC) E-referral System.

This retrospective cross-sectional study analyzed nationwide electronic referral data from the SMARC system between January 2023 and December 2024. The SMARC platform serves as a centralized coordination system for all healthcare referrals between secondary and tertiary care facilities across Saudi Arabia [3,11]. This system manages the entire referral workflow, including request submission, hospital selection assistance, acceptance processing, and transfer coordination through a unified digital infrastructure connecting all governmental and the majority of private hospitals nationwide. To ensure swift access to healthcare services, the SMARC e-referral system provides different types of referrals tailored to patients’ medical conditions (Figure 1). Emergency and routine inpatient referral requests are submitted by the referring hospital into the system and sent to potential receiving hospitals where the requested resources are available. Cases that are not accepted are reviewed by SMARC medical referrals management for triaging and securing an appropriate receiving hospital from a pool of MoH, governmental, and private hospitals within and beyond the referring hospital’s administrative region. Routine outpatient and services, the non-urgent referral types, follow the same pathway where they are submitted by the referring hospital into the SMARC e-referral system and sent to potential receiving hospital offering the required resources. Each referral type has a maximum timeframe for securing acceptance as followed by SMARC: 72 h for emergency, 2 weeks for routine inpatient, and 4 weeks for routine outpatients and services, starting from the initiation of the referral request. Lifesaving referrals are accessed by treating physicians through a 24 h hotline (1937) and are comprehensively reviewed by an on-call consultant to ensure expedited acceptance. Accepted lifesaving requests receive the assigned hospital’s details and are uploaded into the SMARC e-referral system by the referring hospital, while those not accepted are advised to be uploaded into the e-referral system with the recommended referral type.

The study encompassed all 13 administrative regions of Saudi Arabia, organized into five business units (BUs) according to the Kingdom’s healthcare transformation model: Central (Riyadh and Al-Qassim), Eastern, Western (Makkah, Madinah, and Al-Baha), Northern (Al-Jouf, Northern Borders, Tabuk, and Hail), and Southern (Asir, Jazan, and Najran) [11]. Data for this study was extracted from the SMARC database. Data extraction followed standardized protocols with built-in validation checks for completeness and accuracy. As SMARC serves as the mandatory nationwide platform for all healthcare referrals between secondary and tertiary facilities, missing data was very minimal. The study encompasses all MoH participating healthcare institutions across all 13 administrative regions with complete monthly coverage, ensuring comprehensive national representation. The dataset comprised electronic referral records from January 2023 to December 2024, composing of 755,145 referrals across all 13 administrative regions.

### 2.2. Study Variables

The analysis incorporated three main categories of variables: sociodemographic, geographic, and referral-specific characteristics. Sociodemographic variables included patient age (categorized as ≤1, 1–13, 14–17, 18–24, 25–65, and >65 years) and sex. Geographic variables comprised the administrative region of origin and corresponding business unit (BU) within Saudi Arabia’s healthcare transformation model. Referral-specific characteristics included: (1) referral type (routine outpatient department (OPD), emergency, routine inpatient, life-saving, and offsite services where patients temporarily transfer for specialized investigations before returning to their originating facility); (2) bed type requested (OPD) where no bed needed, ward, intensive care unit (ICU), coronary care unit (CCU), neonatal intensive care unit (NICU), pediatric intensive care unit (PICU), isolation, and burn units; (3) reason for referral (subspecialty unavailability, physician unavailability, device unavailability, bed unavailability, health crisis involving disaster response situations requiring coordinated patient allocation, and social reasons including family reunification and proximity considerations); (4) medical specialty involved; (5) referral direction (internal—within the same region, or external—to facilities in different regions); (6) acceptance status (accepted vs. rejected); and (7) temporal indicators (month and year).

### 2.3. Statistical Analysis

Analysis included descriptive statistics (frequencies, percentages) and population-adjusted metrics (referrals per 10,000) calculated using 2023 General Authority for Statistics data [16]. Bivariate relationships were assessed using chi-square tests (significance: *p* < 0.05). Visualizations included line graphs for monthly referral patterns and bar charts for specialty distribution. Analyses were performed using Stata Statistical Software version 17 (StataCorp, 2021, College Station, TX, USA).

### 2.4. Ethical Considerations

This study received ethical approval from the Institutional Review Board of the Saudi Ministry of Health (protocol code 23-77-E and date of approval 20 September 2023). The dataset was fully de-identified prior to analysis, containing no personal identifiers that could link data to individual patients. Data extraction, storage, and analysis followed strict confidentiality protocols, with access restricted to authorized research team members. The requirement for individual informed consent was waived by IRB due to the retrospective analysis of secondary data with no direct patient interaction. All procedures adhered to the principles of the Declaration of Helsinki governing medical research involving human data.

## 3. Results

Analysis of 755,145 e-referrals through the SMARC system during 2023–2024 revealed significant distribution patterns across demographic groups and regions (Table 1). Adults aged 25–65 years comprised the majority of referrals (52.80%), followed by pediatric patients aged 1–13 years (19.10%) and elderly patients over 65 years (13.75%). Notably, elderly patients and infants showed disproportionately high utilization, with referral rates of 1352.87 and 637.23 per 10,000 population, respectively, far exceeding their population representation (2.39% and 1.54%). Sex distribution was relatively balanced (51.47% male), though females demonstrated a higher referral rate (293.25 per 10,000) compared to males (197.52 per 10,000) despite comprising a smaller proportion of the population (38.84% vs. 61.16%). Regional analysis showed the Western BU generated the highest volume of referrals (26.78%), followed by the Southern BU (24.43%). However, when adjusted for population size, the Southern BU showed the highest referral rate (458.69 per 10,000), while the Central BU had the lowest (169.74 per 10,000). Referral distribution showed a slight increase in 2024 compared to 2023 (50.62% vs. 49.38%), with rates of 118.79 and 115.90 per 10,000 population, respectively. Most referrals were managed within their originating administrative regions, with 87.52% being internal referrals (205.41 per 10,000 population) and only 12.48% requiring external referrals to facilities in different regions (29.28 per 10,000 population).

**Figure 2:**
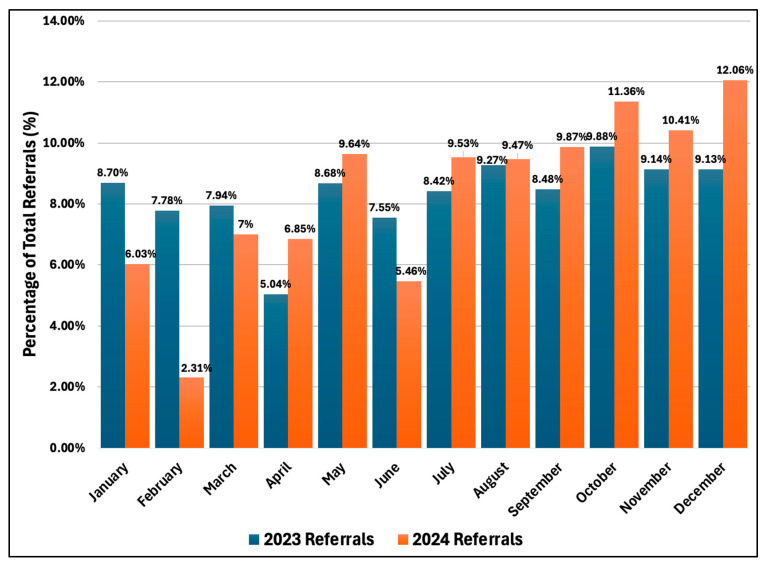
Pattern of percentage monthly e-referrals requests for the years 2023 and 2024 across Saudi Arabia: comparison between 2023 and 2024.

Figure 2 shows the monthly trends in percentage of referrals coordinated through the e-referral system across Saudi Arabia during 2023–2024. The two lines illustrate distinct seasonal patterns and year-over-year differences. In 2023, referral percentages remained relatively stable, ranging from 5.04% to 9.88%, with a noticeable decrease in April (5.04%) and peak in October (9.88%). The distribution showed moderate month-to-month variation with a slight upward trend in the fourth quarter. The 2024 pattern showed more pronounced fluctuations, starting with a steep decline from January (6.03%) to February (2.31%), followed by significant increase in March (7.00%). The middle months showed variable but generally higher percentages compared to 2023, with May reaching 9.64%. The final quarter of 2024 exhibited the highest referral volumes with steadily increasing percentages from October (11.36%) through December (12.06%).

**Figure 3:**
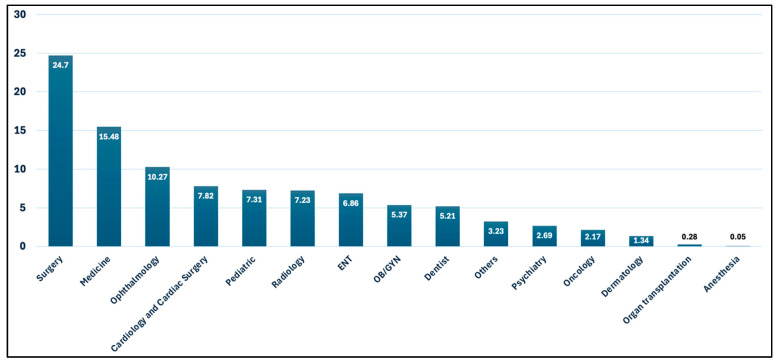
Percentage Distribution of E-Referrals by Medical Specialty across Saudi Arabia (2023–2024).

Figure 3 displays the percentage distribution of e-referrals across medical specialty in Saudi Arabia during 2023–2024. The most common specialties were Surgery, accounting for 24.70% of referrals, followed by Medicine (15.48%) and Ophthalmology (10.27%). Less frequent referrals were for Cardiology and Cardiac Surgery, Pediatrics, Radiology, ENT, Obstetrics/Gynecology, and Dentistry ranging from 5 to 8%. The least common were organ transplantation and anesthesia.

Table 2 shows the distribution of e-referrals in Saudi Arabia during 2023–2024. Routine OPD referrals dominated at 66.66%, followed by emergency (18.59%) and routine inpatient (11.25%). OPD beds accounted for 67.33% of requests, ward beds 22.47%, and critical care beds (ICU, CCU, NICU, and PICU) collectively accounted for 9.91%. Unavailability of subspecialty services was the main referral reason (57.34%), followed by physician unavailability (19.81%) and device unavailability (15.76%).

Table 3 shows e-referral acceptance rates in Saudi Arabia during 2023–2024 were high overall (90.19%) but varied by region and referral characteristics (*p* < 0.0001). Referrals originated from the Eastern (95.65%) and Western (92.17%) BUs had the highest acceptance rates, while the Central BU was lowest (85.43%). At the regional level, referrals originated from Madinah (96.49%) and Northern Borders (96.48%) were highest and Riyadh lowest (84.68%). Life-saving referrals had 100% acceptance. Emergency and offsite services referrals had higher acceptance than routine. NICU and CCU had the highest bed acceptance rates while OPD was lowest. Health crisis and bed unavailability reasons had higher acceptance than social reasons. Internal referrals were more likely to be accepted than external (91.21% vs. 83.04%).

Table 4 shows the distribution of internal and external e-referral destinations by BU and region in Saudi Arabia during 2023–2024. Overall, 87.52% of referrals were internal and 12.48% external. However, destination patterns varied significantly by region and referral characteristics (*p* < 0.0001). The Northern BU had the highest external referrals (30.23%), especially Hail (43.33%) and Al-Jouf (41.55%). The Central BU showed strong internal capacity (93.19%), particularly Riyadh (95.00%). Offsite services referrals were nearly all internal (99.03%), while routine inpatient required the most external transfers (18.26%). NICU and PICU had higher external rates than other beds. Health crisis referrals required more external transfers (32.22%) than other reasons. Rejected referrals were more likely external than accepted ones.

## 4. Discussion

The study aimed to identify pattern and performance of referral overtime in SMARC and identified associated factors that driving referral patterns and outcomes by analyzing regional variations, performance indicators, and progress toward greater self-sufficiency. The SMARC e-referral system demonstrated effective implications of e-health solutions, enhancing coordination between healthcare facilities and securing timely access to healthcare services, in both rural and urban areas. Our findings revealed significant increase in the e-referral requests compared to 2020–2021 [3,11,12] (Table S1), accompanied by a substantial acceptance rate (90.19%), reflecting the effectiveness of the investments made in expanding healthcare workforce and infrastructure within the Saudi healthcare system. Most referrals were managed internally, highlighting optimized resource allocation and regional self-sufficiency in providing healthcare services. The demand for critical care units along with the lifesaving referrals decreased, compared to previous studies [3,11,12], demonstrating the growth expertise in managing such cases and the expansion of specialized healthcare services. OPD referrals significantly increased, suggesting better stabilization of critical patients and more strategic referral practices. These findings demonstrate the significant progression in Saudi Arabia’s healthcare system and underscore the potential outcomes of healthcare’s initiatives within the Health Sector Transformation Plan, in alignment with the Saudi Vision 2030.

Our analysis demonstrates the crucial role of the e-referral system in coordinating access to healthcare services across the regions of Saudi Arabia. The e-referral system showed an expanded capacity to handle referral requests with more than 750,000 referrals, an increase by 19.34% compared to 2020–2021 [12] (Table S1),. While referral requests in 2020–2021 likely influenced by the national COVID-19 restrictions where non-urgent cases such as elective surgeries and outpatient appointments were postponed [22], the 19.34% increase in 2023–2024 highlights the ongoing improvement of the SMARC system and the efficient use of e-health solutions for nationwide hospital coordination. The pronounced monthly fluctuations observed in 2024 compared to 2023 (Figure 2) may reflect continued COVID-19 healthcare impacts through 2023 [23] and dynamic Vision 2030 transformation initiatives implemented throughout 2024 [24] though, further longitudinal investigation is needed to establish causal relationships over years to assess SMARC performance. This higher referral rate was also linked with an increased acceptance rate, reaching 90.19%, around 16% higher than in 2020–2021 [12]. This improvement reflects the healthcare system expansion in providing the services, likely due to the health initiatives under the Health Sector Transformation Plan, which have enhanced both healthcare resources and workforce [24]. According to the MoH statistical yearbook, several infrastructure advancements were successfully implemented between 2021 and 2023, including newly operated hospitals and specialized care centers, and expanding bed capacity and workforce [25,26]. These findings align with the goals of the Health Sector Transformation Plan, which focus on enhancing both access and quality of healthcare services [27].

The demand for critical care services (ICU, CCU, NICU, PICU) reduced collectively from 12.39% in 2020–2021 to 9.91% in 2023–2024, reflecting enhanced healthcare resources in hospitals for managing complex cases [12]. Although this reduction may partly be influenced by the spike in COVID-19 cases in 2020–2021 which required more ICU admission [28,29], it highlights the impact of the MoH’s initiatives to improve healthcare system in Saudi Arabia [27]. The MoH introduced a new Model of Care (MOC) which operates through six integrated care systems: Preventive Care, Urgent Care, Elective Care, Safe Childbirth, Chronic Disease Care, and Palliative Care, which could have alleviated the pressure on critical care resources [30]. This new MOC improved the healthcare system by enhancing chronic disease management (e.g., diabetes) and facilitating early detection of critical conditions including colon, rectal, and breast cancer, thereby reducing the need for critical care units [30]. Also, since 2021, significant efforts have been made to expand critical care services, resulting in more critical care beds in MoH, governmental, and private hospitals [25,26]. Critical care workforce also noticed improvement from 2021 to 2023, including critical care specialists for adults, pediatrics, and neonates [25,26]. These improved parameters are key in managing critically ill patients, potentially reducing hospital’s dependency on external resources to treat such cases [31,32]. The enhanced capability of the hospitals to treat critically ill patients were also reflected in the findings where requests for lifesaving referrals sharply decreased along with the emergency referrals [12]. This decline, particularly in lifesaving cases, reflects the growing expertise in managing these conditions, concurrent with expanded specialized services and resources [25,26]. This has enabled more patients to be stabilized within hospitals and referred for specialized care after the acute phase has passed. Meanwhile, OPD cases significantly increased, indicating improved case management protocols, better stabilization of critical patients, and more strategic referral practices, ultimately supporting the goal of healthcare cost reduction while maintaining the high standards of care.

Referral destination analysis revealed high internal referrals compared to external ones (87.52% and 12.48%, respectively), reflecting the regions’ capability to manage cases independently. Compared to 2020–2021, there has been a reduction for external referrals by 7.39%, indicating improved healthcare resource allocation [3]. Western and Northern BUs showed a notable improvement in managing the cases internally, with 9.54% and 18.42% reduction in external referrals, respectively [3]. However, the Northern BU continues to have the highest external referral requests, suggesting a need for further investigation into factors including population health and healthcare access. Given that health transformation initiatives have recently been implemented, comprehensive evaluation of population health outcomes, resources, and infrastructure in these regions compared to other areas would provide evidence-based foundations for targeted policy interventions and strategic resource allocation decisions. This will provide insights to understand the drivers behind the high referral volume and to identify opportunities for care improvement, thereby reducing the need for external resources. While the Southern BU showed a slight improvement in external referral reduction (0.79%), it is a positive trend toward greater self-sufficiency. The Central and Eastern BUs still maintain the highest internal referrals, above 90% for both, suggesting sustainability of sufficient healthcare resources despite increased demand due to the population growth rate over the years [33]. This regional self-sufficiency could have benefited from the infrastructure expansion of specialized care centers within the regions. According to the MoH statistical year books [25,26], MoH oncology centers expanded from four centers in 2021 located in four administrative regions, to 18 centers by 2023 covering all the 13 administrative regions. Future studies should evaluate the direct impact of such service expansions on regional referral patterns and cost-effectiveness, providing measurable evidence of investment outcomes across specific specialties and regions. Moreover, MoH cardiac centers increased by two more centers to reach 12 centers across the five BUs, in addition to three more diabetes and endocrinology centers. Primary Healthcare Centers (PHCs) also expanded significantly across the regions, reducing the burden on the secondary and tertiary hospitals, thereby enhancing the health resources utilization [34]. Furthermore, the digital health transformation in Saudi Arabia likely played a key role in reducing the reliance on out of region resources [27]. In 2022, Saudi Arabia launched the largest virtual hospital globally (known as SEHA Virtual Hospital), supporting 170 hospitals with 29 basic specialized health services and more than 70 sub-specialty services [35]. Other initiatives with potential influence include the expansion of mobile medical clinic service to cover all administrative regions, serving more than 400,000 patients in 2023 compared to around 91,500 in 2021 [25,26]. These mobile clinics provide different services including maternal care, chronic disease monitoring, and radiological and laboratory services, reducing the pressure on healthcare system and enhancing preventive care [36,37]. Although it is challenging to measure the specific impact of each initiative on minimizing the need for external referrals, these initiatives demonstrated the potential to collectively improve the self-sufficiency of regions in managing cases internally.

The e-referral system’s performance significantly improved between 2020–2021 and 2023–2024, with increasing acceptance rates by 16.06% [12] and internal referrals by 7.39% [3]. These improvements occurred during Saudi Vision 2030’s comprehensive healthcare transformation strategy, which established interconnected initiatives across multiple domains [19,38]. Following the COVID-19 pandemic, a governmental regulation mandated a more than 50% increase in intensive care unit capacity across Saudi healthcare facilities [39,40], resulting in significant infrastructure expansion [6]. Critical care efficiency has markedly improved, with substantially increased acceptance rates of 90.03–94.36%. This represents significant progress from 2021, when acceptance rates ranged from 77.35 to 83.54% [12], demonstrating progress observed following implementation of the Saudi healthcare transformation strategies [39,40]. The multi-faceted transformation approach encompassed several key drivers. Governance reforms implemented under the New Model of Care framework created an integrated coordination system with clearer accountability structures and standardized workflows between healthcare facilities [17]. These governance changes were supported by new policies that focused on directing resources to areas that needed them most, while also setting up performance measures to encourage facilities to keep high rates of accepting critical care transfers [6]. The expansion of medical subspecialty training programs [18] significantly contributed to the 7.39% increase in internal referrals since 2020–2021 [3] by establishing equitably distributed specialist networks across all regions. This expansion enabled local healthcare facilities to handle more complex cases within their regions, particularly in specialized fields (Table 4). This workforce development initiative aligned with Vision 2030’s broader goals of healthcare self-sufficiency and enhanced clinical capabilities [6]. The transformation of organizational culture fostered cross-institutional collaboration, breaking down traditional boundaries and establishing shared accountability for patient outcomes throughout the healthcare journey [6,17]. This cultural shift aligned institutional values with patient-centered care priorities while establishing a foundation for continuous quality improvement. Using offsite services as a new method has successfully cut down on unnecessary patient transfers by enabling specialized tests to be performed without fully admitting patients to hospitals, with 99.03% of these services managed within the same regions (Table 4). The substantial reduction in life-saving referrals to 2.18% demonstrates improved local capacity for acute care management. This improvement demonstrates successful implementation of the Saudi healthcare transformation strategies, which have strengthened emergency stabilization capabilities while establishing more effective pathways for post-acute specialized care referrals [6,17].

These findings provide valuable insights for policymakers seeking to optimize resource allocation across the Kingdom’s healthcare system, supporting the commitment of MoH to continuous improvement in service distribution and accessibility. The geographical patterns identified can inform evidence-based decision-making regarding specialized workforce placement and infrastructure investments, advancing the healthcare service excellence goals outlined in Vision 2030 transformation strategies [6,7]. Strategic recommendations for system enhancement include expanding specialized workforce distribution across regions and integrating AI tools for referral triage to optimize patient allocation, thereby reducing unnecessary transfers and improving regional self-sufficiency [20]. The Saudi e-referral framework demonstrates how centralized coordination and standardized workflows can enhance resource distribution across geographically diverse regions, offering valuable knowledge transfer opportunities for developing health systems globally [6]. Despite their diversity and specialized requirements, OPD cases have increased markedly, comprising 67.33% of all referrals (Table 2). This arises from improved case management protocols, better stabilization of critical patients, and more strategic referral practices, ultimately supporting healthcare cost reduction goals [17]. While substantial progress has been achieved in referral coordination, ongoing challenges in tracking clinical outcomes necessitate expanding platform capabilities to encompass the complete patient journey. Future research priorities should include longitudinal assessment of e-referral system sustainability, integration of emerging technologies in referral decision support, patient-level analysis to examine individual utilization patterns and their impact on healthcare resource allocation, comprehensive geographical analysis examining regional variations in referral patterns and their underlying demographic, socio-economic, and infrastructure determinants and implementation science approaches to identify contextual factors enabling successful adoption across diverse healthcare settings [21]. Additionally, evaluation efforts should utilize the improved acceptance rates (Table 3) to enhance patient outcomes through standardized metrics that directly connect referral efficiency with clinical quality measures. Given the constraints in obtaining clinical outcomes data as secondary sources lack such information, the establishment of a registry system for clinical outcomes is crucial for directly assessing the impact of the referral system.

### Strengths and Limitations

This study offers significant methodological strengths, including analysis of a large national dataset composing of over 750,000 e-referrals across all 13 administrative regions, providing comprehensive visibility into specialty distribution and regional variations across Saudi Arabia’s healthcare system. The inclusion of two consecutive years of data with population-adjusted metrics enables accurate assessment of healthcare access patterns, while our multidimensional analysis approach provides policymakers with valuable insights for evidence-based resource allocation decisions. Nevertheless, the study’s nationwide e-referral dataset cannot capture the full clinical context influencing healthcare providers’ referral choices, and as a retrospective analysis of secondary data, it cannot establish definitive causal relationships between observed improvements and specific healthcare transformation strategies.

## 5. Conclusions

This nationwide analysis of over 750,000 e-referrals demonstrates significant improvements in Saudi Arabia’s healthcare system performance from 2020–2021 to 2023–2024. Acceptance rates markedly increased across all regions and specialties, indicating substantial expansion of healthcare workforce, infrastructure, and services. The rise in internal referrals highlights growing regional self-sufficiency, aligned with Vision 2030 goals. However, variations persist, with lower performance in Northern regions signaling the need for targeted investments. The decline in critical care referrals reflects strengthened capabilities in managing complex cases. Expanded offsite services prevented unnecessary transfers. These quantifiable indicators demonstrate improvements observed during Saudi Arabia’s multidimensional healthcare transformation strategies. Additional research should assess system sustainability and integrate emerging technologies such as utilizing AI tools to further optimize referral coordination and patient outcomes. Saudi Arabia’s model provides valuable insights for developing integrated e-referral frameworks to enhance equitable access.

## Figures and Tables

**Table 1:** Sociodemographic characteristics, population distribution, and referral request rates per 10,000 population across Saudi Arabia (2023–2024).

Characteristics	Frequency N (%) 755,145 (100.00)	Population N (%) 32,175,224 (100.00)	Rate per 10,000 Population
**Age**			
≤1 year	31,571 (4.18)	495,438 (1.54)	637.23
1–13 years	144,209 (19.10)	6,904,509 (21.46)	208.86
14–17 years	21,758 (2.88)	1,849,514 (5.75)	117.64
18–24 years	55,067 (7.29)	3,450,111 (10.72)	159.61
25–65 years	398,681 (52.80)	18,707,959 (58.14)	213.11
>65 years	103,859 (13.75)	767,693 (2.39)	1352.87
**Sex**			
Male	388,681 (51.47)	19,678,595 (61.16)	197.52
Female	366,464 (48.53)	12,496,629 (38.84)	293.25
**BUs**			
***Central BU***	168,513 (22.32)	9,927,927 (30.85)	169.74
Riyadh Region	132,617 (17.56)	8,591,748 (26.70)	154.35
Al-Qassim Region	35,896 (4.75)	1,336,179 (4.15)	268.65
***Eastern BU***	102,580 (13.58)	5,125,254 (15.93)	200.15
Eastern Region	102,580 (13.58)	5,125,254 (15.93)	200.15
***Western BU***	202,205 (26.78)	10,498,620 (32.62)	192.60
Makkah Region	136,321 (18.05)	8,021,463 (24.93)	169.94
Madinah Region	49,416 (6.54)	2,137,983 (6.64)	231.13
Al-Baha Region	16,468 (2.18)	339,174 (1.05)	485.53
***Northern BU***	97,382 (12.90)	2,601,841 (8.01)	374.28
Al-Jouf Region	26,101 (3.46)	595,822 (1.85)	438.07
Northern Borders Region	20,986 (2.78)	373,577 (1.16)	561.76
Tabuk Region	39,107 (5.18)	886,036 (2.75)	441.37
Hail Region	11,188 (1.48)	746,406 (2.31)	149.89
***Southern BU***	184,465 (24.43)	4,021,582 (12.50)	458.69
Asir Region	79,736 (10.56)	2,024,285 (6.29)	393.89
Jazan Region	77,842 (10.31)	1,404,997 (4.36)	554.04
Najran Region	26,887 (3.56)	592,300 (1.84)	453.94
**Year**			
2023	372,926 (49.38)	32,175,224 (100.00)	115.90
2024	382,219 (50.62)	32,175,224 (100.00)	118.79
**Referral destination**			
Internal	660,926 (87.52)	32,175,224 (100.00)	205.41
External	94,219 (12.48)	32,175,224 (100.00)	29.28
**Total**	**755,145 (100.00)**	**32,175,224 (100.00)**	**234.70**

Note: N: frequency; %: percentage; BU: Business Unit. Source: General Authority for Statistics, Saudi Arabia (2023) [16]; Internal referrals represent referrals within the same healthcare region, while external referrals represent referrals to facilities in different regions.

**Table 2:** Distribution of e-referrals by referral characteristics across Saudi Arabia (2023–2024).

Characteristic	N (%)
**Referral Type**	
Routine OPD	503,385 (66.66)
Emergency	140,398 (18.59)
Routine Inpatient	84,956 (11.25)
Life Saving	16,447 (2.18)
offsite Services	9959 (1.32)
**Bed Type**	
OPD no bed	508,425 (67.33)
Ward	169,644 (22.47)
ICU	35,330 (4.68)
CCU	16,418 (2.17)
NICU	14,394 (1.91)
PICU	8715 (1.15)
Isolation Unit	1580 (0.21)
Burn Unit	639 (0.08)
**Referral Reason**	
Subspecialty Unavailability	432,974 (57.34)
Physician Unavailability	149,610 (19.81)
Device Unavailability	118,985 (15.76)
Bed Unavailability	25,276 (3.35)
Health Crisis	25,137 (3.33)
Social Factors	3163 (0.42)
**Total**	**755,145 (100.00)**

Note: OPD = Outpatient Department; ICU = Intensive Care Unit; CCU = Coronary Care Unit; NICU = Neonatal Intensive Care Unit; PICU = Pediatric Intensive Care Unit; N = Number of referrals; % = Percentage of total referrals. Data collected from the Saudi Medical Appointment and Referral Center (SMARC) system during 2023–2024.

**Table 3:** Acceptance decision by business units, regions, referral type, bed type, and referral reason across Saudi Arabia (2023–2024).

Characteristic	Acceptance N (%)	Rejection N (%)	Total N (%)	*p*-Value
**Business Units and Regions**				<0.0001
***Central BU***	143,962 (85.43)	24,551 (14.57)	168,513 (22.32)	
Riyadh Region	112,301 (84.68)	20,316 (15.32)	132,617 (17.56)	
Al-Qassim Region	31,661 (88.20)	4235 (11.80)	35,896 (4.75)	
***Eastern BU***	98,121 (95.65)	4459 (4.35)	102,580 (13.58)	
Eastern Region	98,121 (95.65)	4459 (4.35)	102,580 (13.58)	
***Western BU***	186,371 (92.17)	15,834 (7.83)	202,205 (26.78)	
Makkah Region	123,667 (90.72)	12,654 (9.28)	136,321 (18.05)	
Madinah Region	47,680 (96.49)	1736 (3.51)	49,416 (6.54)	
Al-Baha Region	15,024 (91.23)	1444 (8.77)	16,468 (2.18)	
***Northern BU***	88,347 (90.72)	9035 (9.28)	97,382 (12.90)	
Al-Jouf Region	22,344 (85.61)	3757 (14.39)	26,101 (3.46)	
Northern Borders Region	20,247 (96.48)	739 (3.52)	20,986 (2.78)	
Tabuk Region	36,059 (92.21)	3048 (7.79)	39,107 (5.18)	
Hail Region	9697 (86.67)	1491 (13.33)	11,188 (1.48)	
***Southern BU***	164,286 (89.06)	20,179 (10.94)	184,465 (24.43)	
Asir Region	67,542 (84.71)	12,194 (15.29)	79,736 (10.56)	
Jazan Region	73,150 (93.97)	4692 (6.03)	77,842 (10.31)	
Najran Region	23,594 (87.75)	3293 (12.25)	26,887 (3.56)	
**Referral Type**				<0.0001
Routine OPD	448,037 (89.00)	55,348 (11.00)	503,385 (66.66)	
Emergency	132,148 (94.12)	8250 (5.88)	140,398 (18.59)	
Routine Inpatient	74,928 (88.20)	10,028 (11.80)	84,956 (11.25)	
Life Saving	16,447 (100.00)	0 (0.00)	16,447 (2.18)	
Offsite Services	9527 (95.66)	432 (4.34)	9959 (1.32)	
**Bed Type**				<0.0001
OPD no bed	452,692 (89.04)	55,733 (10.96)	508,425 (67.33)	
Ward	157,502 (92.84)	12,142 (7.16)	169,644 (22.47)	
ICU	31,808 (90.03)	3522 (9.97)	35,330 (4.68)	
CCU	15,425 (93.95)	993 (6.05)	16,418 (2.17)	
NICU	13,582 (94.36)	812 (5.64)	14,394 (1.91)	
PICU	8059 (92.47)	656 (7.53)	8715 (1.15)	
Isolation Unit	1438 (91.01)	142 (8.99)	1580 (0.21)	
Burn Unit	581 (90.92)	58 (9.08)	639 (0.08)	
**Referral Reason**				<0.0001
Subspecialty Unavailability	389,443 (89.95)	43,531 (10.05)	432,974 (57.34)	
Physician Unavailability	135,880 (90.82)	13,730 (9.18)	149,610 (19.81)	
Device Unavailability	105,595 (88.75)	13,390 (11.25)	118,985 (15.76)	
Bed Unavailability	23,614 (93.42)	1662 (6.58)	25,276 (3.35)	
Health Crisis	23,841 (94.84)	1296 (5.16)	25,137 (3.33)	
Social Factors	2714 (85.80)	449 (14.20)	3163 (0.42)	
**Referral Destination**				<0.0001
Internal	602,851 (91.21)	58,075 (8.79)	660,926 (87.52)	
External	78,236 (83.04)	15,983 (16.96)	94,219 (12.48)	
**Total**	**681,087 (90.19)**	**74,058 (9.81)**	**755,145 (100.00)**	

Note: N = Number of referrals; % = Percentage of referrals; BU: Business Unit; OPD = Outpatient Department; ICU = Intensive Care Unit; CCU = Coronary Care Unit; NICU = Neonatal Intensive Care Unit; PICU = Pediatric Intensive Care Unit. Data collected from the Saudi Medical Appointment and Referral Center (SMARC) system during 2023–2024. *p*-values calculated using Pearson’s Chi-square test, indicating significant differences in acceptance rates across all categories.

**Table 4:** Referral destination by business units and regions across Saudi Arabia (2023–2024).

Characteristic	Internal N (%)	External N (%)	Total N (%)	*p*-Value
**Business Units and Regions**				
***Central BU***	157,043 (93.19)	11,470 (6.81)	168,513 (22.32)	<0.0001
Riyadh Region	125,990 (95.00)	6627 (5.00)	132,617 (17.56)	
Al-Qassim Region	31,053 (86.51)	4843 (13.49)	35,896 (4.75)	
***Eastern BU***	95,044 (92.65)	7536 (7.35)	102,580 (13.58)	
Eastern Region	95,044 (92.65)	7536 (7.35)	102,580 (13.58)	
***Western BU***	183,254 (90.63)	18,951 (9.37)	202,205 (26.78)	
Makkah Region	127,937 (93.85)	8384 (6.15)	136,321 (18.05)	
Madinah Region	42,907 (86.83)	6509 (13.17)	49,416 (6.54)	
Al-Baha Region	12,410 (75.36)	4058 (24.64)	16,468 (2.18)	
***Northern BU***	67,942 (69.77)	29,440 (30.23)	97,382 (12.90)	
Al-Jouf Region	15,257 (58.45)	10,844 (41.55)	26,101 (3.46)	
Northern Borders Region	14,798 (70.51)	6188 (29.49)	20,986 (2.78)	
Tabuk Region	31,547 (80.67)	7560 (19.33)	39,107 (5.18)	
Hail Region	6340 (56.67)	4848 (43.33)	11,188 (1.48)	
***Southern BU***	157,643 (85.46)	26,822 (14.54)	184,465 (24.43)	
Asir Region	64,279 (80.61)	15,457 (19.39)	79,736 (10.56)	
Jazan Region	73,551 (94.49)	4291 (5.51)	77,842 (10.31)	
Najran Region	19,813 (73.69)	7074 (26.31)	26,887 (3.56)	
**Referral Type**				<0.0001
Routine OPD	446,392 (88.68)	56,993 (11.32)	503,385 (66.66)	
Emergency	119,929 (85.42)	20,469 (14.58)	140,398 (18.59)	
Routine Inpatient	69,446 (81.74)	15,510 (18.26)	84,956 (11.25)	
Life Saving	15,297 (93.01)	1150 (6.99)	16,447 (2.18)	
Offsite Services	9862 (99.03)	97 (0.97)	9959 (1.32)	
**Bed Type**				<0.0001
OPD no bed	450,398 (88.59)	58,027 (11.41)	508,425 (67.33)	
Ward	148,148 (87.33)	21,496 (12.67)	169,644 (22.47)	
ICU	27,940 (79.08)	7390 (20.92)	35,330 (4.68)	
CCU	14,742 (89.79)	1676 (10.21)	16,418 (2.17)	
NICU	10,918 (75.85)	3476 (24.15)	14,394 (1.91)	
PICU	6799 (78.01)	1916 (21.99)	8715 (1.15)	
Isolation Unit	1442 (91.27)	138 (8.73)	1580 (0.21)	
Burn Unit	539 (84.35)	100 (15.65)	639 (0.08)	
**Referral Reason**				<0.0001
Subspecialty Unavailability	382,400 (88.32)	50,574 (11.68)	432,974 (57.34)	
Physician Unavailability	136,513 (91.25)	13,097 (8.75)	149,610 (19.81)	
Device Unavailability	100,483 (84.45)	18,502 (15.55)	118,985 (15.76)	
Bed Unavailability	21,848 (86.44)	3428 (13.56)	25,276 (3.35)	
Health Crisis	17,039 (67.78)	8098 (32.22)	25,137 (3.33)	
Social Factors	2643 (83.56)	520 (16.44)	3163 (0.42)	
**Acceptance Condition**				<0.0001
Acceptance	602,851 (91.21)	78,236 (83.04)	681,087 (90.19)	
Rejection	58,075 (8.79)	15,983 (16.96)	74,058 (9.81)	
**Total**	**660,926 (87.52)**	**94,219 (12.48)**	**755,145 (100.00)**	

Note: N = Number of referrals; % = Percentage of referrals; BU: Business Unit (BUs represent the primary administrative level, with individual regions listed beneath each BU); OPD = Outpatient Department; ICU = Intensive Care Unit; CCU = Coronary Care Unit; NICU = Neonatal Intensive Care Unit; PICU = Pediatric Intensive Care Unit. Internal refers to referrals within the same healthcare region, while External refers to referrals to facilities in different regions. Data collected from the Saudi Medical Appointment and Referral Center (SMARC) system during 2023–2024. *p*-values calculated using Pearson’s Chi-square test, indicating significant differences in referral destination patterns across all categories.

## Data Availability

Due to national healthcare data protection policies, the raw datasets used in this study cannot be made publicly available. However, researchers may request access to aggregated or de-identified data through the corresponding author, contingent upon appropriate regulatory approvals and data sharing agreements.

## Supplementary Materials

The following supporting information can be downloaded at: https://www.mdpi.com/article/10.3390/healthcare13161945/s1, Table S1: A Comparison of the Performance of Saudi Medical Appointments and Referrals System Between 2020–2021 and 2023–2024 [3,12].

## Abbreviations

The following abbreviations are used in this manuscript:

AI	Artificial Intelligence
BU	Business Unit
CCU	Coronary Care Unit
ICU	Intensive Care Unit
MOC	Model of Care
MoH	Ministry of Health
NICU	Neonatal Intensive Care Unit
OPD	Outpatient Department
PHC	Primary Healthcare Centers
PICU	Pediatric Intensive Care Unit
SMARC	Saudi Medical Appointments and Referrals Centre
