# Supplementary material for: Saudi Medical Appointments and Referrals Center (SMARC) Performance Dynamic: A Comparative National Analysis of 2023–2024 Against Baseline Metrics
Source: Healthcare (Basel). 2025 Aug 8;13(16):1945. doi: 10.3390/healthcare13161945 (PMC12385655; doi:10.3390/healthcare13161945)
Supplement: Supplementary file 1 [file healthcare-13-01945-s001.zip › healthcare-3780927-supplementary.pdf]

Table S1. A comparison of the Performance of Saudi Medical Appointments and Referrals System (SMARC) Between 2020-2021 and 2023-2024

Characteristic	2020-2021 N (%)	2023-2024 N (%)	Compared reference
Total referral requests	632,763	755,145	[1]
Referral acceptance	469,073 (74.13)	681,087 (90.19)	[1]
Referral for critical care services (ICU, CCU, NICU, PICU)	78373 (12.39)	74857 (9.91)	[1]
Acceptance rate of critical care services (ICU, CCU, NICU, PICU)	(77.35-83.54)	(90.03-94.36)	[1]
Life saving referral	42,087 (6.65)	16,447 (2.18)	[1]
Emergency referral	204,589 (32.33)	140,398 (18.59)	[1]
OPD referral	302,815 (47.86)	503,385 (66.66)	[1]
Internal referral	517,182 (80.13)	660,926 (87.52)	[2]
External referral	128,246 (19.87)	94,219 (12.48)	[2]
Western BU external referral	42,701 (18.91)	18,951 (9.37)	[2]
Northern BU external referral	55,281 (48.65)	29,440 (30.23)	[2]
Southern BU external referral	21,008 (15.33)	26,822 (14.54)	[2]
Central BU external referral	5129 (5.24)	11,470 (6.81)	[2]
Eastern BU external referral	4127 (5.80)	7,536 (7.35)	[2]

Note: N = Number of referrals; % = Percentage of referrals; BU: Business Unit; OPD = Outpatient Department; ICU = Intensive Care Unit; CCU = Coronary Care Unit; NICU = Neonatal Intensive Care Unit; PICU = Pediatric Intensive Care Unit. Internal refers to referrals within the same healthcare administrative region, while External refers to referrals to facilities in different administrative regions. Data were collected from the Saudi Medical Appointments and Referrals Center (SMARC) system during 2023-2024 for the current study, and during 2020-2021 from previous studies examining SMARC system performance [1,2].

References:

1. Alharbi, A.A.; Aljerian, N.A.; Binhotan, M.S.; Alghamdi, H.A.; AlOmar, R.S.; Alsultan, A.K.; Arafat, M.S.; Aldhabib, A.; Aloqayli, A.I.; Alwahbi, E.B.; et al. Acceptance of electronic referrals across the Kingdom of Saudi Arabia: results from a national e-health database. *Front Public Health* **2024**, *12*.
2. Aljerian, N.A.; Alharbi, A.A.; Alghamdi, H.A.; Binhotan, M.S.; AlOmar, R.S.; Alsultan, A.K.; Arafat, M.S.; Aldhabib, A.; Alabdulaali, M.K. External Vs Internal e-Referrals: Results from a Nationwide Epidemiological Study Utilizing Secondary Collected Data. *Risk Management and Healthcare Policy* **2024**, 739-751.
